# Hepatic metastasis of gastric cancer is associated with enhanced expression of ethanolamine kinase 2 via the p53–Bcl-2 intrinsic apoptosis pathway

**DOI:** 10.1038/s41416-021-01271-7

**Published:** 2021-02-03

**Authors:** Takashi Miwa, Mitsuro Kanda, Dai Shimizu, Shinichi Umeda, Koichi Sawaki, Haruyoshi Tanaka, Chie Tanaka, Norifumi Hattori, Masamichi Hayashi, Suguru Yamada, Goro Nakayama, Masahiko Koike, Yasuhiro Kodera

**Affiliations:** Department of Gastroenterological Surgery (Surgery II), Nagoya University Graduate School of Medicine, Nagoya, Japan

**Keywords:** Gastric cancer, Gene expression

## Abstract

**Background:**

Gastric cancer (GC) with hepatic metastasis has a poor prognosis. Understanding the molecular mechanisms involved in hepatic metastasis may contribute to the development of sensitive diagnostic biomarkers and novel therapeutic strategies.

**Methods:**

We performed transcriptome analysis of surgically resected specimens from patients with advanced GC. One of the genes identified as specifically associated with hepatic metastasis was selected for detailed analysis. GC cell lines with knockout of the candidate gene were evaluated in vitro and in vivo. Expression of the candidate gene was analysed in GC tissues from 300 patients.

**Results:**

Ethanolamine kinase 2 (*ETNK2*) was differentially upregulated in GC patients with hepatic metastasis. *ETNK2* expression was elevated in GC cell lines derived from haematogenous metastases. *ETNK2* knockout significantly suppressed proliferation, invasion, and migration; increased apoptosis; reduced Bcl-2 protein expression; and increased phosphorylated p53 expression. In mouse xenograft models, *ETNK2* knockout virtually abolished hepatic metastasis. Stratification of GC patients based on *ETNK2* mRNA level revealed significant associations between high *ETNK2* tumour expression and both hepatic recurrence and worse prognosis.

**Conclusions:**

Upregulation of *ETNK2* in GC enhances hepatic metastasis, possibly via dysregulation of p53**–**Bcl-2**-**associated apoptosis. *ETNK2* expression may serve as a biomarker for predicting hepatic recurrence and a therapeutic target.

## Background

Gastric cancer (GC) is one of the leading causes of cancer-related death worldwide.^1^ Despite advances in multimodal therapy, recurrence after curative gastrectomy is common, and the 5-year survival rate for patients with advanced GC is 20–40%.^2,3^ This is due in large part to a combination of frequent diagnosis at an advanced stage, lack of curative therapies, and a paucity of sensitive biomarkers for predicting recurrence. Tumour progression and metastasis are affected by the anatomical site of initial occurrence as well as hemodynamic parameters. Cancers of the gastrointestinal (GI) tract, such as colon cancer, often develop hepatic metastases because venous drainage of the GI tract occurs via the liver portal vein.^4^ In contrast, peritoneal dissemination is more common than hepatic metastasis in GC, despite similar portal vein reflux.^3,5^ In the past few decades, while the incidence of GC declined steadily especially in diffuse-type GC with a preference for peritoneal dissemination, intestinal-type GC located in junction or cardia with a preference for haematogenous metastasis as represented by hepatic metastasis has been increasing relatively.^6,7^

Several recently developed therapeutic strategies have the potential to enhance the prognosis of patients with GC with peritoneal dissemination.^8,9^ In contrast, patients with hepatic metastasis of GC have a dismal prognosis, even though only those harbouring a single and small metastatic site have acceptable outcomes.^10,11^ The development of therapeutic strategies for hepatic metastasis of GC has stalled; therefore, there is an urgent need to identify molecules specifically involved in hepatic metastasis of GC. The development of hepatic metastases involves many processes, including enhancement of cell adhesion, migration, invasion, and survival, coupled with changes enabling evasion of the immune system.^12^ Differential expression of a diverse array of molecules contribute to those metastatic processes, suggesting the possibility of identifying hepatic metastasis-specific molecules.

To this end, we performed transcriptome analysis of surgically resected GC specimens to identify molecules specifically associated with hepatic metastasis. One of the identified candidate genes, ethanolamine kinase 2 (*ETNK2*), was confirmed to be specifically overexpressed in primary GC tissues from patients with hepatic recurrence. We performed an in-depth analysis of GC cell lines with *ETNK2* knockout (KO) and/or overexpression in vitro and in vivo and additionally analysed *ETNK2* expression in GC tissue specimens and evaluated its potential utility as a biomarker of hepatic metastasis in GC patients.

## Methods

### Clinical specimens and analysis

Resected GC tissues were obtained between 2006 and 2013 from 16 patients who underwent curative gastrectomy for pStage III GC followed by S-1 adjuvant monotherapy and had no recurrences for >5 years (*n* = 4, group 1), liver-confined recurrences within 2 years after surgery (*n* = 4, group 2), peritoneal recurrences within 2 years after surgery (*n* = 4, group 3), and distant nodal recurrences within 2 years after surgery (*n* = 4, group 4). Before sequencing, those samples were satisfied by the following two quality checks: the optical density of extracted RNA was measured to confirm that the ratio of the absorbance at 260 and 280 nm ranged from 1.8 to 2.0, and RNA integrity number measured by Agilent 2100 Bioanalyzer (Agilent Technologies, Santa Clara, CA, USA) was ≥8.0. RNA libraries were prepared by purification of amplicons with AMPure XP beads (Beckman Coulter, Brea, CA, USA), and transcriptome analysis was performed using the HiSeq platform (Illumina, San Diego, CA, USA).^13^ Samples were subjected to global expression profiling of 57,749 genes and significantly differentially expressed genes were selected based on log2-fold change and *P* value using the Cuffdiff package (Homo sapiens Ensemble GRCh37, Ensemble reference 75).

For large-scale analysis of the selected gene expression, primary GC tissues were collected from 300 patients who underwent gastrectomy for GC at the Department of Gastroenterological Surgery, Nagoya University Hospital between 2001 and 2017. Uncommon histologic type, like hepatoid carcinoma, were excluded. Immediately after resection, fresh tissue samples were frozen in liquid nitrogen and stored at −80 °C until analysis. Relevant clinical data were retrieved from a prospectively compiled departmental database. Written informed consent was obtained from all patients, as required by the Institutional Review Board of Nagoya University, Japan (approval no. 2014-0043).

### Cell lines

GC cell lines, the differentiated type (AGS, IM95, MKN1, MKN7, MKN74 and N87) and the undifferentiated type (GCIY, KATO-III, MKN45, NUGC2, NUGC3, NUGC4, OCUM1 and SC-6-JCK), were obtained from the Japanese Collection of Research Bio Resources Cell Bank (Osaka, Japan) or the American Type Culture Collection (ATCC, Manassas, VA, USA). A control, non-tumorigenic epithelial cell line (FHs 74) was purchased from the ATCC. The cells were cultured at 37 °C in RPMI medium (Thermo Fisher Scientific, Waltham, MA, USA) supplemented with 10% foetal bovine serum in an atmosphere containing 5% CO_2_. All cell lines were authenticated using the short tandem repeat PCR method by the Japanese Collection of Research Bio Resources Cell Bank before the study commenced.

### Quantitative reverse-transcription PCR (qRT-PCR) and PCR array analysis

*ETNK2* mRNA levels were determined by qRT-PCR as previously described^14^ using the ABI StepOnePlus Real-Time PCR System (Applied Biosystems, Foster City, CA, USA). Glyceraldehyde-3-phosphate dehydrogenase (*GAPDH*) was amplified as an endogenous control. Specific primer sequences are listed in Table S1. A PCR array analysis was performed to obtain the data that support the involvement of *ETNK2* in epithelial–mesenchymal transition (EMT) by identifying cancer-related genes expressed simultaneously with *ETNK2*. We used the Human Epithelial to Mesenchymal Transition RT2 Profiler PCR Array (Qiagen, Hilden, Germany) to analyse 84 gene expression levels that encode proteins with the functions related to EMT in 14 GC cell lines and *ETNK2* KO cell line. Genes that fulfilled the following criteria were considered as co-ordinately expressed genes with *ETNK2*: (1) expressing at levels with correlation coefficients ≥0.65 with *ETNK2*, and (2) downregulated in the *ETNK2* KO cells compared to the control MKN1 cells.

### Clustered, regularly interspaced, palindromic repeats-associated (CRISPR)/Cas9 editing and generation of stable *ETNK2* KO cell lines

We employed genome editing using the cRISPR-Cas9 method to establish GC cells lines with stable *ETNK2* KO as described previously.^15,16^ Briefly, a guide RNA (gRNA) complementing the sequences flanking *ETNK2* exon 1 was designed using the Gene Art CRISPR gRNA Design Tool (Thermo Fisher Scientific) and synthesised using a Gene Art Precision gRNA Synthesis Kit (Thermo Fisher Scientific). The gRNA (240 ng) was incubated with 1 µg of Gene Art Platinum Cas9 nuclease (Thermo Fisher Scientific) at room temperature and then introduced into GC cells via electroporation using a Neon System (Thermo Fisher Scientific). After evaluating cleavage efficiencies according to the fragmentation patterns on agarose gel electrophoresis, single-cell clones were isolated using a standard limiting dilution method. Cells with stable *ETNK2* KO were selected and KO was confirmed by DNA sequencing and western blot analysis. The sequences of the gRNA target and primers used to detect *ETNK2* cleavage are described in Table S1.

### Transient *ETNK2* knockdown (KD) with small interfering RNA (siRNA)

GC cells were cultured in 24-well plates at 2.5 × 10^4^ cells/well for 24 h and then transiently transfected by addition of 20 pmol *ETNK2*-specific siRNA or a control siRNA (Table S1) combined with LipoTrust EX Oligo (Hokkaido System Science, Sapporo, Japan), as described previously.^14^ After transfection, cells were cultured in serum-free RPMI medium for 72 h before use in experiments.

### Transient *ETNK2* overexpression

GC cells were incubated in 6-well plates at 1 × 10^4^ cells/well and transfected by addition of 4 µg pCMV6-Entry *ETNK2* expression vector (NM_018208; OriGene, Rockville, MD, USA) or a control pCMV6-Entry Tagged Cloning Vector with C-terminal Myc-DDK Tags (PS100001; OriGene) combined with LipoTrust EX Gene (Hokkaido System Science).^17^ After transfection, cells were cultured at 37 °C in RPMI medium for 48 h before use in experiments.

### Cell proliferation, adhesion, invasion, and migration assays

Proliferation of parental GC cells or cells with *ETNK2* KO, *ETNK2* KD, or *ETNK2* overexpression were analysed using the Cell Counting Kit-8 (CCK-8) assay (Dojindo Molecular Technologies, Inc., Kumamoto, Japan). Adhesion to the extracellular matrix proteins fibronectin, collagens I and IV, fibrinogen, and laminin I was examined using a CytoSelect 48-Well Cell Adhesion Assay Kit (Cell Biolabs, Inc., San Diego, CA, USA). Cell invasion was assessed using BioCoat Matrigel invasion chambers (BD Biosciences, Bedford, MA, USA), and migration was assessed using a wound-healing assay as described previously.^14^

### Cell cycle analysis

GC cell cycle analysis was performed using a Cell-Clock Cell Cycle Assay (Biocolor, Carrickfergus, UK) according to the manufacturer’s protocol.^18^ Pixel colour detection and counting of labelled cells were quantified using the ImageJ software (National Institutes of Health, Bethesda, MD, USA). To test the reproducibility of the cell crock assay, we evaluated influences of *ETNK2* KO on cell cycle regulation using the Muse Cell Cycle Kit (Merck Millipore, Billerica, MA, USA) under the manufacturer’s protocol.

### Apoptosis assays

Apoptotic cells were detected by staining with annexin V-Alexa Fluor 568 conjugate (A13202, Thermo Fisher Scientific).^19^ Briefly, parental or stable *ETNK2* KO GC cell lines (1 × 10^5^ cells/ml) were mixed with 10 µl of annexin V conjugate and incubated for 15 min. Cells irradiated with ultraviolet light for 120 min served as a positive control. The cells were visualised by phase contrast and fluorescence microscopy using a BZ9000 microscope (Keyence, Osaka, Japan). To quantify the percentage of cell apoptosis, the total number of cells and annexin V-positive cells in eight randomly selected fields were counted. To measure mitochondrial transmembrane potential and total caspase activity, 5.0 × 10^4^ cells/condition were collected and analysed using a Muse MitoPotential Kit or a Muse MultiCaspase Kit (Merck Millipore, Billerica, MA, USA), respectively, according to the manufacturer’s protocols.^20,21^

### Western blot analysis and Simple Western assays

ETNK2 and B cell lymphoma 2 (Bcl-2) were analysed by traditional western blot analysis. In brief, GC cells were lysed with RIPA buffer and protein concentrations were determined. Samples equivalent to 20 µg of total protein were subjected to sodium dodecyl sulfate-polyacrylamide gel electrophoresis and transferred to polyvinylidene difluoride membranes as previously described.^22^ Blots were probed with mouse anti-ETNK2 polyclonal antibody (LC-C1790607; LSBio, Seattle, WA, USA) diluted 1:100 or rabbit anti-Bcl-2 monoclonal antibody (ab32124; Abcam, Cambridge, UK) diluted 1:100 and then with anti-mouse or anti-rabbit IgG, horseradish peroxidase-linked antibody as secondary antibodies (#7076 and #7074; Cell Signaling Technology, Tokyo, Japan). Blots were developed with Can Get Signal Solution (NKB-101; TOYOBO, Osaka, Japan). β-Actin was used for an endogenous control.

Signal transducer and activator of transcription 3 (Stat3), Bcl-2 associated agonist of cell death (Bad), and p53 were analysed using Simple Western assays (ProteinSimple, San Jose, CA, USA) using a Jess Protein Normalisation Separation Module (ProteinSimple) according to the manufacturer’s protocol.^23^ Briefly, 6 µg of total protein was loaded and probed with the following primary antibodies at 1:50 dilution (all monoclonal antibodies; Cell Signaling Technology): rabbit anti-Stat3 (#12640), rabbit anti-phosphorylated (p)-Stat3 (P-Tyr705) (#9145), rabbit anti-Bad (#9239), rabbit anti-p-Bad (P-Ser122) (#5284), rabbit anti-p53 (#2527), and rabbit anti-p-p53 (P-Ser15) (#9284). Protein expression levels were normalised to total protein and the data were evaluated using the Compass for Simple Western software (ProteinSimple).

### Mouse xenograft model

The Animal Research: Reporting of In Vivo Experiments guidelines were followed for all animal experiments,^24^ and the study was approved by The Animal Research Committee of Nagoya University (IRB no. 29329). Six-week-old male nude mice (BALB/cSlc-nu/nu) were obtained from Chubu Kagaku Shizai (Nagoya, Japan) and mice were housed at least 1 week before experiments in temperature-controlled rooms with a free access to water supply. Parental or stable *ETNK2* KO GC cell lines (1 × 10^6^ cells each) were resuspended in 50 µl of phosphate-buffered saline (PBS) plus 50 µl Matrigel (BD Biosciences) and subcutaneously injected into both flanks of the mice (*n* = 6/group).^16^ Tumour growth was measured every week, and the mice were sacrificed at 8 weeks after injection. Approximate tumour volumes (mm^3^) were calculated as *d*^2^ × *D*/2, where *d* and *D* are the shortest and longest diameters, respectively. Immunohistochemical (IHC) analysis was performed using formalin-fixed subcutaneous tumour to evaluate ETNK2 expression and status of apoptosis (cleaved caspase-3, cleaved poly ADP-ribose polymerase (PARP)) and hypoxia (hypoxia-inducible factor-1a (HIF-1a)) in the tumours. The antibodies used were as follows: ETNK2 (LC-C1790607; LSBio, diluted 1:500), cleaved caspase-3 (#9664; Cell Signaling Technology, diluted 1:500), cleaved PARP (#5625; Cell Signaling Technology, diluted 1:50), and HIF-1a (20960–1-AP, ProteinTech Inc., Manchester, UK, diluted 1:150).

To evaluate hepatic metastasis of GC tumours, 6-week-old male Nod-SCID mice (nod/shi-SCID) were purchased from Japan SLC, Inc. (Hamamatsu, Japan), and mice were housed at least 1 week before experiments in temperature-controlled rooms with a free access to water supply. Mice were treated under general anaesthesia using isoflurane and laparotomised. Then parental or stable *ETNK2* KO cell lines (5 × 10^5^ cells each) were resuspended in 100 µl of PBS and injected directly into the portal vein of the mice (*n* = 4/group) using a 35-gauge needle. After injection of the cell suspensions, we oppressed the puncture site of the portal vein for haemostasis. The mice were imaged using an In Vivo Imaging System (IVIS) Lumina system (Xenogen, Alameda, CA, USA) every 4 weeks after injection, and the volumes of hepatic metastases were measured. To visualise tumours, mice were injected with d-luciferin (150 mg/kg; Summit Pharmaceuticals International, Tokyo, Japan) intraperitoneally and luciferase activity was measured 15 min later using the IVIS. Living Image version 2.6 software (Xenogen) was used to acquire and analyse the data. As a second method to detect metastasis formation, we examined mice by magnetic resonance imaging (MRI; MRS 3000; MR solutions, Guildford, UK) at 12 weeks after GC cell injection, and the mice were then sacrificed.^25^ Mice were euthanised by CO_2_ exposure for 5 min and were observed for 20 min after confirmation of respiration cease.

### IHC staining

Surgically resected specimens from 88 patients with Stage II–III GC were stained for ETNK2 as described previously.^26^ In brief, formalin-fixed, paraffin-embedded sections were incubated for 1 h at room temperature (25 °C) with a mouse anti-ETNK2 polyclonal antibody (LC-C1790607; LSBio) diluted 1:250. The level of ETNK2 protein expression was graded depending on the percentage of stained cells as follows: negative (0%), weak (≤20%), and strong (20%<). Specimens were randomised, coded, and analysed by two independent observers blinded to the clinical data. All specimens were evaluated at least twice within a given time interval by both observers to minimise intra-observer variation.

### External validation data

Kaplan–Meier Plotter (KM plotter) (http://kmplot.com/analysis/) and The Cancer Genome Atlas (TCGA) were used to obtain GC data sets for external validation of our institutional data.^27,28^ The TCGA data set was analysed for overall survival and the KM plotter data set was analysed for both overall and disease-free survival.

### Statistical analysis

Qualitative and quantitative variables were compared using Fisher’s exact test and Mann–Whitney test, respectively. To evaluate the paired bivariate correlation, we employed the Spearman’s rank correlation coefficient. We employed the KM method to evaluate the survival curves. Differences in survival, hazard ratios (HRs), and 95% confidence intervals (CIs) were calculated using the Cox proportional hazards models. Risk factors and odds ratios (ORs) for hepatic metastasis and recurrence were evaluated using logistic regression analysis. Cumulative recurrence rates were analysed using Grey test. All analyses were performed using the R software (The R Foundation for Statistical Computing, Vienna, Austria) and EZR software (Saitama Medical Center, Jichi Medical University, Saitama, Japan).^29^ *P* < 0.05 was considered significant.

## Results

### *ETNK2* is overexpressed in primary GC tissues from patients with hepatic recurrence

We performed transcriptome analysis of GC tissues from patients who underwent curative gastrectomy for pStage III GC and experienced no recurrence for ≥5 years or experienced hepatic, peritoneal, or distal nodal recurrence within 2 years of surgery. Among the 57,749 genes analysed, 23 molecules with high expression were identified exclusively in the hepatic recurrence group (Table 1). Among them, we decided to focus on *ETNK2* for the following reasons: it showed high specificity for hepatic recurrence indicated by little changes in the peritoneal and nodal recurrence groups, the nucleotide sequence of *ETNK2* is available (NM_001297760.2), and there were no previous reports about association between *ETNK2* and cancers of the GI tract. Furthermore, we performed pilot experiments to determine a target molecule and confirm reproducible results of the transcriptome data in 23 molecules with high expression identified exclusively in the hepatic recurrence group. We first examine mRNA expression levels in cell lines to test whether the gene is overexpressed in GC cell lines. Next, we forwarded to the expression analysis using clinical tissues from 78 selected GC patients whose prognosis and recurrence pattern are known to test whether the expression levels of the gene is likely to have correlation with hepatic recurrence of GC (data not shown). Through these processes, we selected *ETNK2* as a target molecule of this study.

**Table 1:** List of genes overexpressed in gastric cancer tissues from patients with hepatic recurrence within 2 years after curative gastrectomy.

Symbol	H-rec/Non-rec		Full name	Function	Location	Localisation	P-rec/Non-rec	N-rec/Non-rec
	Log_2_ FC	*P*					Log_2_ FC	Log_2_ FC
*ETNK2*	2.421	<0.001	Ethanolamine kinase 2	Ethanolamine phosphorylation	1q32.1	Cytosol	−0.606	1.390
*FABP3*	3.774	<0.001	Fatty acid-binding protein 3	Fatty acid transporter	1p35.2	Extracellular space	0.051	0.992
*TCF7L1*	2.288	<0.001	Transcription factor 7 like 1	Transcription factor	2p11.2	Nucleus, cytosol	−0.260	−0.386
*FRAS1*	2.319	<0.001	Fraser extracellular matrix complex subunit 1	Cell adhesion	4q21.21	Extracellular space	0.587	1.586
*RNF182*	5.362	<0.001	Ring finger protein 182	E3 ubiquitin-protein ligase	6p23	Nucleus	−0.124	2.317
*CYP2W1*	6.809	<0.001	Cytochrome P450 family 2 subfamily W member 1	Catalytic activity	7p22.3	Endoplasmic reticulum	1.450	1.667
*PRSS1*	4.203	<0.001	Protease, serine 1	Serine protease	7q34	Extracellular space	0.122	0.952
*RBP4*	3.549	<0.001	Retinol-binding protein 4	Carrier for retinol	10q23.33	Extracellular space	−1.186	0.834
*GAL*	4.278	<0.001	Galanin and GMAP prepropeptide	Neuroendocrine peptide	11q13.2	Extracellular space	2.076	1.340
*HMGA2*	3.291	<0.001	High mobility group AT-hook 2	Transcriptional regulator	12q14.3	Nucleus	0.421	0.612
*ASGR2*	3.560	<0.001	Asialoglycoprotein 2	Mediator of endocytosis of plasma glycoproteins	17p13.1	Plasma membrane	−0.124	0.452
*SMTNL2*	4.739	<0.001	Smoothelin like 2	Unknown	17p13.2	Nucleus	−0.879	1.108
*COMP*	4.187	<0.001	Cartilage oligomeric matrix protein	Extracellular matrix protein	19p13.11	Extracellular space	0.760	1.173
*BCAM*	2.123	<0.001	Basal cell adhesion molecule	Laminin receptor	19q13.32	Plasma membrane	−0.554	0.042
*HIF3A*	4.168	<0.001	Hypoxia inducible factor 3 alpha subunit	Regulator of hypoxia-inducible genes	19q13.32	Nucleus, cytosol	0.290	−0.018
*TNNT1*	3.316	<0.001	Troponin T1, slow skeletal type	Regulator of striated muscle contraction	19q13.42	Cytoskeleton, cytosol	1.675	−0.637
*GATA5*	2.944	<0.001	GATA-binding protein 5	Transcription factor	20q13.33	Nucleus	−1.401	−0.398
*HIC2*	3.434	<0.001	HIC ZBTB transcriptional repressor 2	Transcriptional repressor	22q11.21	Nucleus	0.523	0.843
*SUSD2*	2.976	<0.001	Sushi domain containing 2	Cytokine receptor	22q11.23	Plasma membrane	0.464	0.302
*MYO18B*	4.731	<0.001	Myosin XVIIIB	Regulator of muscle specific genes	22q12.1	Cytoskeleton	4.325	−0.660
*GPC3*	2.990	<0.001	Glypican 3	Multifunction membrane protein	Xq26.2	Plasma membrane	−0.997	0.465
*IGSF1*	3.546	<0.001	Immunoglobulin superfamily member 1	Immunoglobulin	Xq26.2	Plasma membrane	−0.899	0.601
*TKTL1*	6.109	<0.001	Transketolase like 1	Transketolase	Xq28	Nucleus	−2.080	−2.758

*Log-FC* log fold change, *H-rec* hepatic recurrence, *Non-rec* non-recurrence, *P-rec* peritoneal recurrence, *N-rec* nodal recurrence.

### *ETNK2* is highly expressed in GC cell lines derived from haematogenous metastases and correlates with genes associated with the EMT

**Fig. 1:**
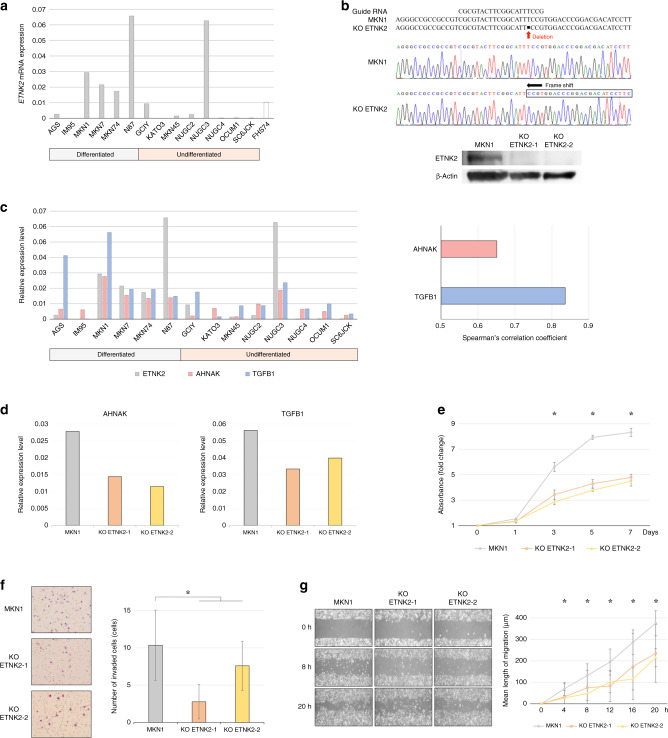
*ETNK2* is upregulated in human GC cell lines and promotes malignant behaviours. **a** qRT-PCR analysis of *ETNK2* mRNA levels in human GC cell lines. **b** CRISPR/Cas9-mediated knockout (KO) of *ETNK2* in MKN1 cells. Upper panel: DNA sequencing of the PCR product encompassing the *ETNK2* exon indicates successful base deletion in MKN1 KO cells. Lower panel: western blot analysis of ETNK2 protein expression in two MKN1 *ETNK2* KO cell lines. **c** Spearman’s correlation analysis of 84 cancer-related genes with *ETNK2* in GC cell lines. The levels of *AHNAK* and *TGFB1* significantly correlate with that of *ETNK2*. **d** The fold changes of transcripts identified in PCR array analysis in *ETNK2* KO cells. **e** Proliferation of parental and *ETNK2* KO MKN1 cell lines. **f** Images (left) and quantification (right) of Matrigel invasion assays of parental and *ETNK2* KO MKN1 cells. **g** Images (left) and quantification (right) of wound-healing migration assays of parental and *ETNK2* KO cell lines. *P < 0.005. Data are presented as the mean ± standard deviation.

We analysed the expression levels of *ETNK2* mRNA in a panel of six differentiated and eight undifferentiated human GC cell lines (Fig. 1a). While the expression levels varied considerably, MKN1, MKN7, MKN74, N87, and NUGC3 cell lines expressed higher levels of *ETNK2* than FHS74. To facilitate analysis of *ETNK2* function in GC cells, we used the MKN1 cell line for genome editing, because it was originally derived from a liver metastasis lesion from a GC patient, expressed one of the highest levels of *ETNK2* mRNA, had high abilities in cell migration and invasion in our previous studies, and is engrafted in nude mice for subcutaneous and in Nod-SCID mice for hepatic metastasis xenograft models.^25,30^ We generated two MKN1 cell lines with stable *ETNK2* KO (KO ETNK2-1 and ETNK2-2) using the CRISPR-Cas9 method. Cleavage was confirmed by agarose gel electrophoresis (Fig. S1a) and DNA sequencing (Fig. 1b), which revealed a single base-pair deletion resulting in a frame shift in the *ETNK2* coding sequence. Consistent with this, ETNK2 protein expression was undetectable by western blot analysis (Fig. 1b). When we determined the expression levels of 84 EMT-related genes, we found that the mRNAs encoding AHNAK nucleoprotein (*AHNAK*) and transforming growth factor beta 1 (*TGFB1*) were expressed at levels that correlated significantly with those of *ETNK2* mRNA (Fig. 1c). *ETNK2* KO cell lines expressed lower levels of *AHNAK* and *TGFB1* than MKN1 cells (Fig. 1d).

### *ETNK2* expression modulates the malignant behaviour of GC cell lines

**Fig. 2:**
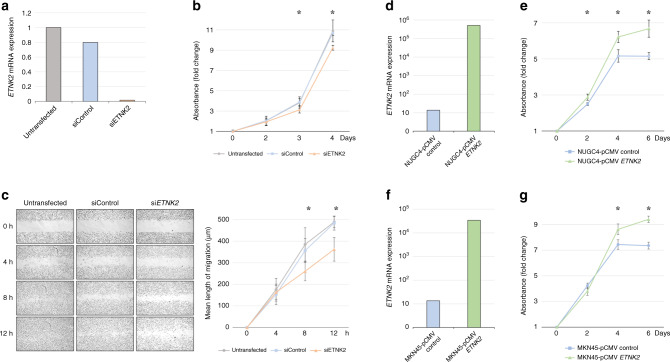
*ETNK2* knockdown and overexpression influence the proliferation and migration of GC cells. **a** qRT-PCR analysis. **b** Proliferation assay of untransfected, control siRNA-transfected, and *ETNK2* siRNA-expressing MKN1 cells. **c** Images (left) and quantification (right) of wound-healing migration assays of untransfected, control siRNA-transfected, and *ETNK2* siRNA-expressing MKN1 cells. **d** qRT-PCR analysis. **e** Proliferation assays of NUGC4 GC cells expressing control pCMV6 or pCMV6-*ETNK2* expression vector. **f** qRT-PCR analysis. **g** Proliferation assays of MKN45 cells expressing control pCMV6 or pCMV6-*ETNK2* expression vector. *P < 0.005. Data are presented as the mean ± standard deviation.

Next, we examined the effects of *ETNK2* KO on MKN1 cell proliferation, invasion, and migration in vitro. We found that all three properties were significantly reduced compared with the unmanipulated parental MKN1 cell line (Fig. 1e–g). Similarly, *ETNK2* KO MKN1 cells showed a slightly reduced ability to adhere to collagen I and collagen IV but not to the other matrix proteins tested, compared with the parental cell line (Fig. S1b). To confirm these findings, we transiently silenced or overexpressed *ETNK2* in GC cells by transfection with *ETNK2*-targeting siRNA or an *ETNK2* expression vector, respectively. We found that *ETNK2* KD also decreased the proliferation and migration of MKN1 cells (Fig. 2a–c), consistent with the effects of stable *ETNK2* KO. Moreover, forced expression of *ETNK2* in NUGC4 and MKN45 cells, which expressed low *ETNK2* mRNA levels (Fig. 1a), had the opposite effect and enhanced the proliferation of both cell lines (Fig. 2d–g).

### *ETNK2* KO induces apoptosis and cell cycle arrest in GC cell lines

**Fig. 3:**
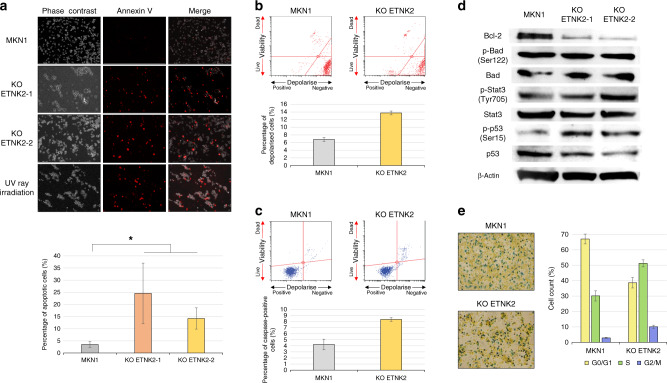
*ETNK2* knockout promotes cell cycle arrest and apoptosis of GC cells. **a** Fluorescence and phase contrast micrographs of untransfected and *ETNK2* KO MKN1 cells stained with annexin V (red) to detect apoptotic cells. Irradiated cells served as a positive control. **b** Flow cytometric dot plots (upper) and quantification (lower) of untransfected and *ETNK2* KO MKN1 cells stained to detect cells with mitochondrial membrane potential depolarisation. **c** Flow cytometric dot plots (upper) and quantification (lower) of untransfected and *ETNK2* KO MKN1 cells stained with anti-caspase antibody. **d** Western blot analysis of apoptosis-related proteins in untransfected and *ETNK2* KO MKN1 cells. β-Actin served as a loading control. **e** Light micrographs (left) and quantification (right) of untransfected and *ETNK2* KO MKN1 cells stained with Redox dye to detect cells in G0/G1, S, and G2/M phases of the cell cycle. *P < 0.005. Data are presented as the mean ± standard deviation.

To determine how *ETNK2* KO inhibits cell proliferation, we first examined apoptosis using an annexin V assay. We found that the MKN1 cell lines with stable *ETNK2* KO exhibited increased annexin V staining compared with parental MKN1 cells (Fig. 3a). *ETNK2* KO also caused an increase in mitochondrial membrane potential depolarisation (Fig. 3b) and caspase activity (Fig. 3c), which are both consistent with induction of the intrinsic mitochondrial pathway of apoptosis. Moreover, western blot analysis revealed decreased expression of the anti-apoptotic protein Bcl-2 in *ETNK2* KO MKN1 cells compared with parental cells (Fig. 3d), whereas Simple Western assays revealed no effect of *ETNK2* KO on the expression of Bad, p-Bad (Ser122), Stat3, and p-Stat3 (Tyr705). (Fig. 3d). Notably, however, *ETNK2* KO increased the expression of the phosphorylated form of p53 (Ser15) but not of total p53 (Fig. 3d). Accordingly, a decrease of the number of cells in G0/G1 phase and an increase of the number of cells in G2/M phase were exhibited in *ETNK2* KO cells compared to the control MKN1 cells (Fig. 3e and Fig. S1c), indicative of cell cycle delay or arrest.

### *ETNK2* promotes the growth and hepatic metastasis of GC cells in a mouse xenograft model

**Fig. 4:**
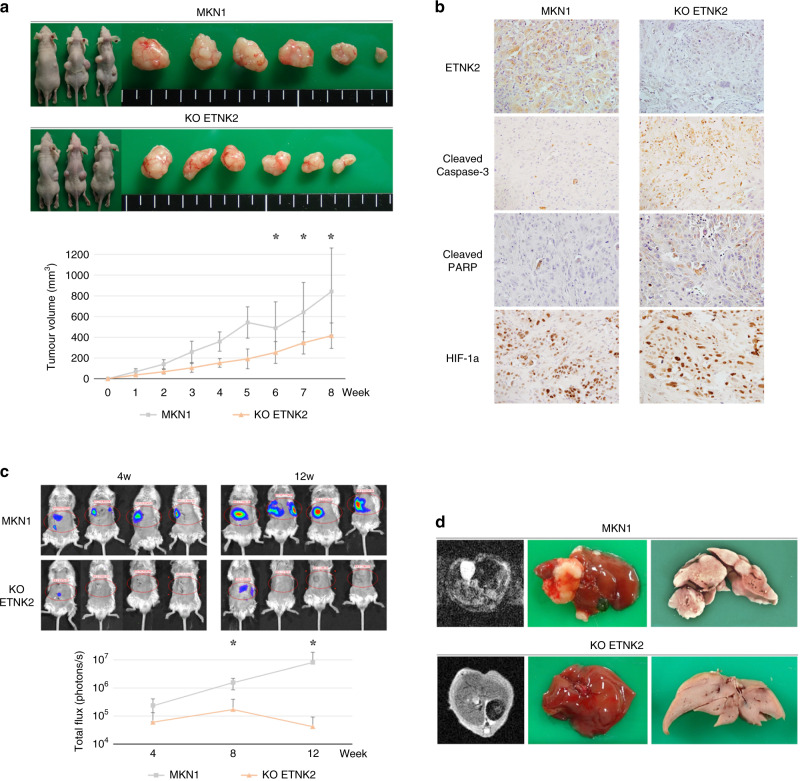
*ETNK2* knockout reduces the growth and hepatic metastasis of GC cells in a mouse xenograft model. **a** Images of mice and excised tumours (upper) and quantification of tumour volumes (lower) after subcutaneous injection of mice with untransfected or *ETNK2* KO MKN1 cells. **b** Results of immunohistochemical analysis of ETNK2, cleaved caspase-3, cleaved PARP, and HIF-1a in subcutaneous tumours formed by parental MKN1 cells and *ETNK2* KO cells. **c** In vivo bioluminescent imaging of hepatic metastases (upper) and quantification of the bioluminescence signal in mice injected with untransfected or *ETNK2* KO MKN1 cells (lower). **d** MRI and macroscopic image of the liver in mice injected with untransfected or *ETNK2* KO MKN1 cells. *P < 0.005. Data are presented as the mean ± standard deviation.

To determine whether our in vitro findings on the behaviour of GC cells are also observed in vivo, we first examined the effects of *ETNK2* KO on the growth of MKN1 cells after subcutaneous injection in BALB/c nude mice. Indeed, the *ETNK2* KO cells exhibited significantly decreased growth compared with parental MKN1 cells (Fig. 4a). In IHC analysis, we found loss of ETNK2 expression in subcutaneous tumours from *ETNK2* KO cells. Furthermore, we found increased expression of cleaved caspase-3 and cleaved PARP in subcutaneous tumours from *ETNK2* KO cells compared to those from a control MKN1 cells. In contrast, no differences in HIF-1a expression were observed (Fig. 4b). To examine hepatic metastasis, we injected Nod-SCID mice with parental or *ETNK2* KO MKN1 cells expressing a luciferase reporter and monitored the luminescence signals by whole-animal in vivo imaging. We observed that mice bearing *ETNK2* KO tumours emitted significantly weaker luminescence signals compared with the parental tumours, and no hepatic metastasis could be detected by MRI imaging (Fig. 4c, d). At 12 weeks after cell injection, the macroscopic appearance of liver specimens from mice injected with parental MKN1 cells revealed multiple tumour nodules, whereas none were detected in the livers of mice implanted with the *ETNK2* KO cell line (Fig. 4d).

### High *ETNK2* expression in GC tissues is associated with hepatic recurrence and poor prognosis

**Fig. 5:**
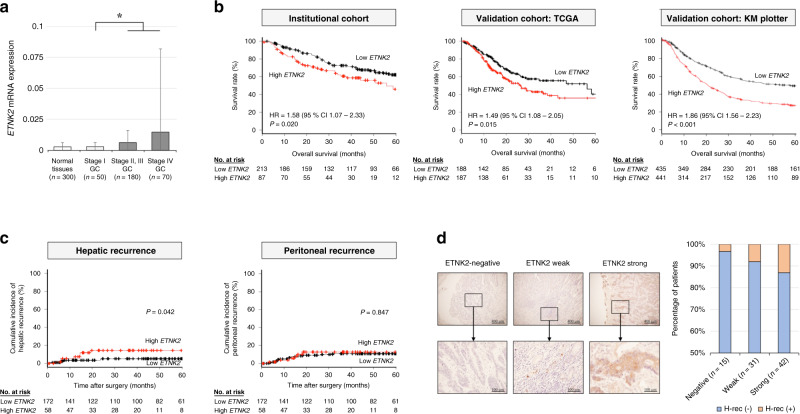
*ETNK2* mRNA expression in clinical GC tissues is significantly associated with hepatic recurrence and prognosis. **a** qRT-PCR analysis of *ETNK2* mRNA levels in normal and GC tissues from patients in our institutional cohort according to disease stage. **b** Kaplan–Meier overall survival curves for patients with Stage I–IV GC in the institutional and validation cohorts. **c** Cumulative incidence of hepatic and peritoneal recurrence in patients with Stage I–III GC in the institutional cohort. **d** IHC staining of GC specimens from patients in our institutional cohort. Left panels show representative images of tissues categorised as negative, weak, and strong staining for ETNK2 protein. Right panel shows ETNK2 expression in patients with and without haematogenous recurrence (*n* = 88). Data in **a** are presented as the mean ± standard deviation.

To assess the clinical significance of *ETNK2* mRNA expression in GC, we first analysed its expression in data sets from institutional cohort, consisting of normal stomach tissues and GC specimens from patients with Stage I, II/III, or IV GC. *ETNK2* mRNA was present at significantly higher levels in the more advanced stages of GC (II–IV) (Fig. 5a). We then performed receiver operating characteristic curve analysis to examine the ability of *ETNK2* mRNA expression to predict disease recurrence within 5 years of curative gastrectomy in a 300-patient cohort from our institution, which gave a cut-off value of 0.006 for *ETNK2* mRNA (Fig. S1d). Subsequently, we stratified the 300 patients into high (*n* = 87) and low (*n* = 213) *ETNK2* mRNA expression groups based on the cut-off value. The clinicopathological characteristics of the two groups are shown in Table S2. High *ETNK2* expression was significantly associated with vessel invasion, lymph node metastasis, and disease stages. Analysis of KM survival curves showed a significant association between high *ETNK2* mRNA and significantly shorter overall survival in the full institutional cohort (*n* = 300, HR 1.58, 95% CI 1.07–2.33, *P* = 0.020; Fig. 5b). Disease-free survival in the Stage II/III GC patients subset tended to be short but not significant (*n* = 180, HR 1.43, 95% CI 0.83–1.43, *P* = 0.203; Fig. S1e). To validate our institutional data, we also analysed GC patient data sets from TCGA and KM plotter databases. High *ETNK2* expression was significantly associated with worse overall survival in both the TCGA data set (HR 1.49, 95% CI 1.08–2.05, *P* = 0.015) and KM plotter data set (HR 1.86, 95% CI 1.56–2.23, *P* < 0.001) (Fig. 5b), and high expression was additionally associated with worse disease-free survival in the KM plotter data set (HR 1.59, 95% CI 1.21–2.10, *P* < 0.001) (Fig. S1e). Of note, the cumulative incidence of hepatic recurrence, but not of peritoneal recurrence, was also significantly higher in the high versus low *ETNK2* expression group in the institutional data set (Fig. 5c). Finally, multivariable analysis identified high *ETNK2* mRNA expression as an independent risk factor for hepatic metastasis and/or hepatic recurrence (Table S3).

### ETNK2 protein expression in GC tissues is associated with haematogenous recurrence

Finally, we also examined the relationship between ETNK2 protein expression and recurrence by IHC staining of GC tissues from our institutional cohort of 88 patients with Stage II/III GC. Although there were only a few cases with ETNK2 staining at the fundic gland region of the stomach, no stained cells were found in epithelial cells and stromal tissues. Figure 5d and Fig. S1f show the typical staining patterns for classification of negative, weak, or strong ETNK2 staining intensity. We found that a higher proportion of patients with haematogenous recurrence exhibited positive ETNK2 expression (weak or strong staining) in GC tissue compared with patients without haematogenous recurrence (Fig. 5d).

## Discussion

In this study, we conducted pattern-specific transcriptome analysis of GC tissues to identify molecules potentially involved in hepatic metastasis. One of the genes, *ETNK2*, was specifically upregulated in GC tissues from patients with hepatic recurrence after curative gastrectomy, suggesting a possible causative link. We established stable *ETNK2* KO GC cell lines and demonstrated a role for *ETNK2* in behaviours associated with metastasis, namely, enhanced proliferation, migration, and invasion and reduced apoptosis. Compared to FHs74 cell, four out of five GC cell lines established from haematogenous metastatic tissues had higher expression levels of *ETNK2*, supporting our hypothesis that *ETNK2* promotes hepatic metastasis. We also examined *ETNK2* expression at the mRNA and protein levels in surgically resected GC specimens and identified significant positive associations between high expression and worse prognosis and hepatic recurrence. Thus *ETNK2* expression in GC tissues may have potential utility as a biomarker for predicting hepatic recurrence.

The *ETNK2* gene is located on human chromosome 1q32.1, and the gene product is ubiquitously expressed in human tissues. *ETNK2* is a member of the choline/ethanolamine kinase family and catalyses the first step in the cytidine diphosphate ethanolamine pathway. This enzyme plays a role in the biosynthesis of phosphatidylethanolamine, a main constituent of cell membranes.^31^ Only a few previous reports of association between *ETNK2* and malignancies and one report suggested that a higher level of CpG methylation in the *ETNK2* promoter was related to radiotherapy resistance in laryngeal squamous cell carcinoma.^32^ However, in general, little is known about the function of *ETNK2* in GI tract cancers, including GC.

Phosphatidylethanolamine is abundant in mitochondria, and its depletion has been shown to induce apoptosis via changes in mitochondrial morphology and fragmentation in mammalian cells.^33,34^ Additionally, cell apoptosis is an important process to develop distant metastasis and regulated by multiple stimuli, for example, loss of adhesion to extracellular matrix by invasion and migration (anoikis), hypoxia in the circulation, and DNA damage by chemotherapy.^35–37^ Based on these previous reports, we investigated the involvement of *ETNK2* in apoptosis. We hypothesised that *ETNK2* may have anti-apoptotic effects and that *ETNK2* KO would also affect the malignant phenotypes of GC cells. Consistent with this, we confirmed that *ETNK2* KO promoted apoptosis and cell cycle arrest and attenuated the behaviours required for distant metastasis formation (proliferation, invasion, and migration). The extrinsic pathway of apoptosis is activated by binding of ligands to cell surface death receptors,^38^ whereas the intrinsic pathway is induced by mitochondrial membrane depolarisation resulting from opening of the mitochondrial permeability transition pore. Cytochrome c is released from the mitochondrial matrix into the cytosol, where it activates caspases, the ‘executioners’ of apoptosis.^39,40^ One of the consequences of caspase activation is the induction of DNA fragmentation, a hallmark of apoptosis.^41^ In the present study, *ETNK2* KO was associated with enhanced mitochondrial membrane depolarisation and caspase activation in GC cell lines. Bcl-2 controls the mitochondrial membrane permeability and inhibits caspase activity by preventing the release of cytochrome c from the mitochondria.^42,43^ We observed that *ETNK2* KO decreased Bcl-2 protein expression in GC cell lines, while Bad and Stat3 phosphorylation were unaffected. Interestingly, phosphorylation of p53 was increased by *ETNK2* KO. Bad inhibits Bcl-2 activity, while Stat3 and p53 induce Bcl-2 transcription.^44–46^ Activation of p53 by phosphorylation represses Bcl-2 transcription. Thus these results support a role for *ETNK2* in the Bcl-2**-**associated intrinsic pathway of apoptosis via p53 phosphorylation.

Cell cycle progression is regulated by a series of checkpoints, failure of which can lead to cell cycle arrest, inhibition of proliferation, and induction of apoptosis.^47^ We found that *ETNK2* KO decreased the proportion of cells in the G0/G1 phase. Activated p53 induces transcription of p21, an inhibitor of the checkpoint regulatory protein cyclin-dependent kinase-1 and -2,^48^ suggesting another mechanism by which *ETNK2* affects the GC cell cycle and proliferation. Taken together, our results suggest that *ETNK2* may have anti-apoptotic effects in GC cells via direct or indirect regulation of p53 phosphorylation, leading to enhanced proliferation, invasion, and migration, culminating in hepatic metastasis formation.

EMT is a process in which cells lose their epithelial properties and gain migration and invasive ability to become mesenchymal cells, which plays an important role in cancer metastasis.^49^ We conducted PCR array analysis to search for EMT-related genes whose expression is correlated with *ETNK2*. Consequently, we found that *ETNK2* mRNA expression levels positively correlated to those of *AHNAK* and *TGFB1*. Activated *TGFB1* phosphorylates Smad2 and Smad3 proteins. These Smad proteins activated by phosphorylation acts as transcription factors by assembling with Smad4 and regulates cell proliferation, migration, and differentiation.^50^ *AHNAK* has diverse role as oncogene or tumour-suppressor gene.^51,52^ *AHNAK* promotes EMT via TGFB/Smad signalling pathway and regulates cell migration and metastasis.^53^ Additionally, we revealed lower expression of *AKNAK* and *TGFB1* in *ETNK2* KO cell lines. Our results indicate that *ETNK2* acted as an upstream mediator of *AHNAK* signalling and downstream target of *TGFB1* in its signalling pathway.

We confirmed our in vitro findings using a mouse xenograft model of GC. Both the tumorigenicity and ability to form hepatic metastases were strikingly reduced by *ETNK2* KO; indeed, hepatic metastasis was virtually abolished. We also found increased expression of cleaved caspase-3 and cleaved PARP in *ETNK2* KO subcutaneous tumours by IHC analysis. In contrast, subcutaneous tumours formed by both parental MKN1 and *ETNK2* KO cells have no differences in the expression of HIF-1a, which mediates the cellular response to hypoxia as transcriptome factor.^54^ Caspase-3 is an effector caspase that is cleaved and activated by initiator caspase. The activated caspase-3 induces apoptosis, as a result, PARP are cleaved by caspase-3 during apoptosis.^55^ These findings suggest the involvement of *ETNK2* in cell apoptosis in vivo. Because hepatic metastasis was modelled here by directly injecting parental or *ETNK2* KO GC cells into the portal vein of the mice, our results strongly support a role for *ETNK2* in promoting hepatic metastasis formation, which is likely to be mediated by a reduction in apoptosis and/or enhancement of cell survival during portal vein reflux and/or invasion and growth within the liver microenvironment.

We found that patients with high *ETNK2* mRNA levels in clinical GC samples was significantly associated with vessel invasion, lymph node metastasis, and advanced disease stage with poor prognosis. Our results indicated that *ETNK2* contributes, at least in part, to cancer progression via lymphatic systems. On the other hand, the cumulative incidence of hepatic recurrence was significantly higher in patients with high *ETNK2* expression, whereas peritoneal recurrence was not influenced by *ETNK2* mRNA expression. Moreover, high *ETNK2* mRNA expression was also an independent risk factor for hepatic metastasis and hepatic recurrence, supporting our hypothesis that *ETNK2* preferentially promotes hepatic metastasis in GC. Between hepatic metastasis and peritoneal dissemination, there are differences in the microenvironment around cancer cells, such as hetero aggregates containing and premetastatic niche in circulating tumour cell, lymphatic orifices on the peritoneal surface, and human peritoneal mesothelial cells altered by stimulation with a number of growth factors in peritoneal-free cancer cell.^56,57^ *ETNK2* may promote hepatic metastasis by inducing anti-apoptotic effects and EMT in such a tumour microenvironment that is suitable specifically for hepatic metastasis formation. Similarly, detection of ETNK2 protein expression by IHC staining could also be helpful in predicting hepatic recurrence after curative gastrectomy. Of note, IHC is a simple and frequently used procedure in clinical settings. Patients identified to have high tumour expression of ETNK2 could undergo aggressive postoperative surveillance using enhanced MRI or ultrasonography to ensure early detection of hepatic recurrence.

Current evidence supports the importance of multimodal therapy for advanced GC. Although S-1 monotherapy as postoperative adjuvant chemotherapy for advanced GC has shown little success in suppressing haematogenous recurrence, more aggressive adjuvant doublet chemotherapy has been beneficial.^58,59^ However, aggressive chemotherapy can have serious adverse effects. Therefore, using ETNK2 expression as a biomarker for hepatic recurrence may enable more individualised selection of appropriate adjuvant chemotherapy regimens for patients undergoing curative resection for GC.

Our study has several limitations. First, p53–Bcl-2**-**mediated apoptosis and malignant phenotypes are required for metastasis to sites other than the liver, including the peritoneal cavity, and we cannot conclude that *ETNK2* specifically promotes hepatic metastasis. In this regard, useful information could be obtained from experiments with co-cultured tumour cells and hepatic sinusoidal endothelial cells/peritoneal mesothelial cells and/or evaluation of orthotopic mouse xenograft models. Second, we identified *ETNK2* by transcriptome analysis of patients with hepatic recurrence who underwent curative gastrectomy for pStage III GC followed by S-1 adjuvant monotherapy. Because many anti-cancer drugs induce apoptosis, it is possible that *ETNK2* is associated with drug resistance. Although such data were not available for this study, they will contribute to a better understanding of the role of *ETNK2* in GC. Finally, assays to detect ETNK2 expression in serum samples would greatly advance the possible clinical applications of our findings.^60^

In conclusion, this study demonstrated that *ETNK2* promotes hepatic metastasis formation of GC, possibly via dysregulation of the p53**–**Bcl-2**-**associated intrinsic apoptosis pathway and enhancement of malignant phenotypes. *ETNK2* expression in GC tissues may have utility as a biomarker for predicting hepatic recurrence. *ETNK2* and associated signalling pathways may also serve as targets for the development of new therapeutic strategies for the suppression of hepatic recurrence and improvement of the prognosis of patients with advanced GC.

## Supplementary information

Supplementary Materials

## Data Availability

The data that support the findings of this study are available from the corresponding author upon reasonable request.

## References

[1] Jemal A, Bray F, Center MM, Ferlay J, Ward E, Forman D (2011). Global cancer statistics. CA Cancer J. Clin..

[2] Mokadem I, Dijksterhuis WPM, van Putten M, Heuthorst L, de Vos-Geelen JM, Haj Mohammad N (2019). Recurrence after preoperative chemotherapy and surgery for gastric adenocarcinoma: a multicenter study. Gastric Cancer.

[3] Nashimoto A, Akazawa K, Isobe Y, Miyashiro I, Katai H, Kodera Y (2013). Gastric cancer treated in 2002 in Japan: 2009 annual report of the JGCA nationwide registry. Gastric Cancer.

[4] Guraya SY (2019). Pattern, stage, and time of recurrent colorectal cancer after curative surgery. Clin. Colorectal Cancer.

[5] Sakuramoto S, Sasako M, Yamaguchi T, Kinoshita T, Fujii M, Nashimoto A (2007). Adjuvant chemotherapy for gastric cancer with S-1, an oral fluoropyrimidine. N. Engl. J. Med..

[6] Kusano C, Gotoda T, Khor CJ, Katai H, Kato H, Taniguchi H (2008). Changing trends in the proportion of adenocarcinoma of the esophagogastric junction in a large tertiary referral center in Japan. J. Gastroenterol. Hepatol..

[7] Smyth EC, Verheij M, Allum W, Cunningham D, Cervantes A, Arnold D (2016). Gastric cancer: ESMO Clinical Practice Guidelines for diagnosis, treatment and follow-up. Ann. Oncol..

[8] Ishigami H, Kitayama J, Kaisaki S, Hidemura A, Kato M, Otani K (2010). Phase II study of weekly intravenous and intraperitoneal paclitaxel combined with S-1 for advanced gastric cancer with peritoneal metastasis. Ann. Oncol..

[9] Kanda M, Shimizu D, Tanaka H, Tanaka C, Kobayashi D, Hayashi M (2018). Synaptotagmin XIII expression and peritoneal metastasis in gastric cancer. Br. J. Surg..

[10] Kodera Y, Fujitani K, Fukushima N, Ito S, Muro K, Ohashi N (2014). Surgical resection of hepatic metastasis from gastric cancer: a review and new recommendation in the Japanese gastric cancer treatment guidelines. Gastric Cancer.

[11] Oki E, Tokunaga S, Emi Y, Kusumoto T, Yamamoto M, Fukuzawa K (2016). Surgical treatment of liver metastasis of gastric cancer: a retrospective multicenter cohort study (KSCC1302). Gastric Cancer.

[12] Shimizu D, Kanda M, Kodera Y (2018). Emerging evidence of the molecular landscape specific for hematogenous metastasis from gastric cancer. World J. Gastrointest. Oncol..

[13] Kanda M, Shimizu D, Tanaka H, Tanaka C, Kobayashi D, Hayashi M (2018). Significance of SYT8 for the detection, prediction, and treatment of peritoneal metastasis from gastric cancer. Ann. Surg..

[14] Miwa T, Kanda M, Tanaka H, Tanaka C, Kobayashi D, Umeda S (2017). FBXO50 enhances the malignant behavior of gastric cancer cells. Ann. Surg. Oncol..

[15] Liang X, Potter J, Kumar S, Zou Y, Quintanilla R, Sridharan M (2015). Rapid and highly efficient mammalian cell engineering via Cas9 protein transfection. J. Biotechnol..

[16] Umeda, S., Kanda, M., Miwa, T., Tanaka, H., Tanaka, C., Kobayashi, D. et al. Fraser extracellular matrix complex subunit 1 promotes liver metastasis of gastric cancer. *Int. J. Cancer*10.1002/ijc.32705 (2019). 10.1002/ijc.32705 31597194

[17] Ishiwata H, Suzuki N, Ando S, Kikuchi H, Kitagawa T (2000). Characteristics and biodistribution of cationic liposomes and their DNA complexes. J. Control Release.

[18] Sato K, Sato M, Yokoyama M, Hirai M, Furuta A (2019). Influence of culture conditions on cell proliferation in a microfluidic channel. Anal. Sci..

[19] Koopman G, Reutelingsperger CP, Kuijten GA, Keehnen RM, Pals ST, van Oers MH (1994). Annexin V for flow cytometric detection of phosphatidylserine expression on B cells undergoing apoptosis. Blood.

[20] Gollapudi S, McCormick MJ, Gupta S (2003). Changes in mitochondrial membrane potential and mitochondrial mass occur independent of the activation of caspase-8 and caspase-3 during CD95-mediated apoptosis in peripheral blood T cells. Int. J. Oncol..

[21] Riedl SJ, Shi Y (2004). Molecular mechanisms of caspase regulation during apoptosis. Nat. Rev. Mol. Cell Biol..

[22] Shimizu D, Kanda M, Sugimoto H, Shibata M, Tanaka H, Takami H (2017). The protein arginine methyltransferase 5 promotes malignant phenotype of hepatocellular carcinoma cells and is associated with adverse patient outcomes after curative hepatectomy. Int. J. Oncol..

[23] O’Neill RA, Bhamidipati A, Bi X, Deb-Basu D, Cahill L, Ferrante J (2006). Isoelectric focusing technology quantifies protein signaling in 25 cells. Proc. Natl Acad. Sci. USA.

[24] Kilkenny C, Browne WJ, Cuthill IC, Emerson M, Altman DG (2012). Improving bioscience research reporting: the ARRIVE guidelines for reporting animal research. Osteoarthr. Cartil..

[25] Miwa T, Kanda M, Umeda S, Tanaka H, Shimizu D, Tanaka C (2019). Establishment of peritoneal and hepatic metastasis mouse xenograft models using gastric cancer cell lines. In Vivo.

[26] Kanda M, Tanaka H, Shimizu D, Miwa T, Umeda S, Tanaka C (2018). SYT7 acts as a driver of hepatic metastasis formation of gastric cancer cells. Oncogene.

[27] Adam JB, Vesteinn T, Ilya S, Sheila MR, Michael M, Brady B (2014). Comprehensive molecular characterization of gastric adenocarcinoma. Nature.

[28] Szasz AM, Lanczky A, Nagy A, Forster S, Hark K, Green JE (2016). Cross-validation of survival associated biomarkers in gastric cancer using transcriptomic data of 1,065 patients. Oncotarget.

[29] Kanda Y (2013). Investigation of the freely available easy-to-use software ‘EZR’ for medical statistics. Bone Marrow Transplant..

[30] Kanda M, Shimizu D, Sawaki K, Nakamura S, Umeda S, Miwa T (2020). Therapeutic monoclonal antibody targeting of neuronal pentraxin receptor to control metastasis in gastric cancer. Mol. Cancer.

[31] Lykidis A, Wang J, Karim MA, Jackowski S (2001). Overexpression of a mammalian ethanolamine-specific kinase accelerates the CDP-ethanolamine pathway. J. Biol. Chem..

[32] Kim JS, Kim SY, Lee M, Kim SH, Kim SM, Kim EJ (2015). Radioresistance in a human laryngeal squamous cell carcinoma cell line is associated with DNA methylation changes and topoisomerase II alpha. Cancer Biol. Ther..

[33] Steenbergen R, Nanowski TS, Beigneux A, Kulinski A, Young SG, Vance JE (2005). Disruption of the phosphatidylserine decarboxylase gene in mice causes embryonic lethality and mitochondrial defects. J. Biol. Chem..

[34] Vance JE, Tasseva G (2013). Formation and function of phosphatidylserine and phosphatidylethanolamine in mammalian cells. Biochim. Biophys. Acta.

[35] Chen D, Lin X, Gao J, Shen L, Li Z, Dong B (2018). Wee1 inhibitor AZD1775 combined with cisplatin potentiates anticancer activity against gastric cancer by increasing DNA damage and cell apoptosis. Biomed. Res. Int..

[36] Douma S, Van Laar T, Zevenhoven J, Meuwissen R, Van Garderen E, Peeper DS (2004). Suppression of anoikis and induction of metastasis by the neurotrophic receptor TrkB. Nature.

[37] Gilkes DM, Semenza GL, Wirtz D (2014). Hypoxia and the extracellular matrix: drivers of tumour metastasis. Nat. Rev. Cancer.

[38] Harrington, C. T., Sotillo, E., Robert, A., Hayer, K. E., Bogusz, A. M., Psathas, J. et al. Transient stabilization, rather than inhibition, of MYC amplifies extrinsic apoptosis and therapeutic responses in refractory B-cell lymphoma. *Leukemia*10.1038/s41375-019-0454-4 (2019). 10.1038/s41375-019-0454-4 PMC6884148 30914792

[39] Sharma A, Boise LH, Shanmugam M (2019). Cancer metabolism and the evasion of apoptotic cell death. Cancers.

[40] Song, C., Han, Y., Luo, H., Qin, Z., Chen, Z., Liu, Y. et al. HOXA10 induces BCL2 expression, inhibits apoptosis, and promotes cell proliferation in gastric cancer. *Cancer Med.*10.1002/cam4.2440 (2019). 10.1002/cam4.2440 PMC6745829 31364281

[41] Arya, J. S., Joseph, M. M., Sherin, D., Nair, J. B., Manojkumar, T. K. & Maiti, K. K. Exploring mitochondria mediated intrinsic apoptosis by new phytochemical entities: an explicit observation of cytochrome c dynamics on lung and melanoma cancer cells. *J. Med. Chem.*10.1021/acs.jmedchem.9b01098 (2019). 10.1021/acs.jmedchem.9b01098 31393121

[42] Hockenbery D, Nunez G, Milliman C, Schreiber RD, Korsmeyer SJ (1990). Bcl-2 is an inner mitochondrial membrane protein that blocks programmed cell death. Nature.

[43] Miyashita T, Reed JC (1993). Bcl-2 oncoprotein blocks chemotherapy-induced apoptosis in a human leukemia cell line. Blood.

[44] Hemann MT, Lowe SW (2006). The p53-Bcl-2 connection. Cell Death Differ..

[45] Selvakumaran M, Lin HK, Miyashita T, Wang HG, Krajewski S, Reed JC (1994). Immediate early up-regulation of bax expression by p53 but not TGF beta 1: a paradigm for distinct apoptotic pathways. Oncogene.

[46] Xie Q, Yang Z, Huang X, Zhang Z, Li J, Ju J (2019). Ilamycin C induces apoptosis and inhibits migration and invasion in triple-negative breast cancer by suppressing IL-6/STAT3 pathway. J. Hematol. Oncol..

[47] Tay KC, Tan LT, Chan CK, Hong SL, Chan KG, Yap WH (2019). Formononetin: a review of its anticancer potentials and mechanisms. Front. Pharmacol..

[48] Abbas T, Dutta A (2009). p21 in cancer: intricate networks and multiple activities. Nat. Rev. Cancer.

[49] Lamouille S, Xu J, Derynck R (2014). Molecular mechanisms of epithelial-mesenchymal transition. Nat. Rev. Mol. Cell Biol..

[50] Bellomo C, Caja L, Moustakas A (2016). Transforming growth factor β as regulator of cancer stemness and metastasis. Br. J. Cancer.

[51] Lu D, Wang J, Shi X, Yue B, Hao J (2017). AHNAK2 is a potential prognostic biomarker in patients with PDAC. Oncotarget.

[52] Zhao Z, Xiao S, Yuan X, Yuan J, Zhang C, Li H (2017). AHNAK as a prognosis factor suppresses the tumor progression in glioma. J. Cancer.

[53] Sohn M, Shin S, Yoo JY, Goh Y, Lee IH, Bae YS (2018). Ahnak promotes tumor metastasis through transforming growth factor-β-mediated epithelial-mesenchymal transition. Sci. Rep..

[54] Bárdos JI, Ashcroft M (2005). Negative and positive regulation of HIF-1: a complex network. Biochim. Biophys. Acta.

[55] Cohen GM (1997). Caspases: the executioners of apoptosis. Biochem. J..

[56] Gay LJ, Felding-Habermann B (2011). Contribution of platelets to tumour metastasis. Nat. Rev. Cancer.

[57] Sun F, Feng M, Guan W (2017). Mechanisms of peritoneal dissemination in gastric cancer. Oncol. Lett..

[58] Bang YJ, Kim YW, Yang HK, Chung HC, Park YK, Lee KH (2012). Adjuvant capecitabine and oxaliplatin for gastric cancer after D2 gastrectomy (CLASSIC): a phase 3 open-label, randomised controlled trial. Lancet.

[59] Yoshida K, Kodera Y, Kochi M, Ichikawa W, Kakeji Y, Sano T (2019). Addition of docetaxel to oral fluoropyrimidine improves efficacy in patients with stage III gastric cancer: interim analysis of JACCRO GC-07, a randomized controlled trial. J. Clin. Oncol..

[60] Kanda M, Suh YS, Park DJ, Tanaka C, Ahn SH, Kong SH (2020). Serum levels of ANOS1 serve as a diagnostic biomarker of gastric cancer: a prospective multicenter observational study. Gastric Cancer.

