# Supplementary material for: Hepatic metastasis of gastric cancer is associated with enhanced expression of ethanolamine kinase 2 via the p53–Bcl-2 intrinsic apoptosis pathway
Source: Br J Cancer. 2021 Feb 3;124(8):1449–60. doi: 10.1038/s41416-021-01271-7 (PMC8039033; doi:10.1038/s41416-021-01271-7)

|  | **Experiment** | **Type** | **Sequence (5′ - 3′)** | **Product size** | **Annealing temperature** |
| --- | --- | --- | --- | --- | --- |
| ***ETNK2*** | qRT-PCR | forward | TGAAGAACGAGATCAACCCC | 120 bp | 60 °C |
|  |  | reverse | GACAAAACACCACAGGGGAC |  |  |
|  | gRNA | gRNA target sequence | CGCGTACTTCGGCATTTCCG |  | |
|  | Cleavage detection | Cleavage forward | CTGCTGGGAATGGCTGTG | 278 bp | 64 °C |
|  |  | Cleavage reverse | CCACTCCGCTACCTTGGTC |  |  |
|  | siRNA | si*ETNK2*-1 | GGCACAAGAUCGACAAUUATT |  |  |
|  |  | si*ETNK2*-2 | GGCUCUACGUGCAAGUCAATT |  |  |
| ***GAPDH*** | qRT-PCR | forward | GAAGGTGAAGGTCGGAGTC | 226 bp | 60 °C |
|  |  | probe | CAAGCTTCCCGTTCTCAGCC |  |  |
|  |  | reverse | GAAGATGGTGATGGGATTTC |  |  |

**Table S1.** Sequences of oligonucleotides

*ETNK2*, ethanolamine kinase 2; *GAPDH*, glyceraldehyde-3-phosphate dehydrogenase; qRT-PCR, quantitative reverse-transcription PCR; gRNA, guide RNA; siRNA, small interfering RNA.

**Table S2. Association between *ETNK2* mRNA levels and clinicopathological parameters for 300 patients with gastric cancer**

| **Variables** | **High *ETNK2***  **expression (n)** | **Low *ETNK2***  **expression (n)** | ***P*** |
| --- | --- | --- | --- |
| Age  < 65 years  ≥ 65 years | 31  56 | 94  119 | 0.198 |
| Sex  Male  Female | 66  21 | 150  63 | 0.396 |
| Carcinoembryonic antigen (ng/ml)  ≤ 5  > 5 | 66  21 | 173  40 | 0.343 |
| Carbohydrate antigen 19-9 (IU/ml)  ≤ 37  > 37 | 68  19 | 168  45 | 0.878 |
| Tumor location  Entire  Upper third  Middle third  Lower third | 8  23  23  33 | 14  50  73  76 | 0.531 |
| Macroscopic type  Borrmann 1/2/3  Borrmann 4/5 | 73  14 | 186  27 | 0.461 |
| Tumor size (mm)  < 60  ≥ 60 | 57  30 | 121  92 | 0.195 |
| Tumor depth (UICC 8th)  pT1  pT2  pT3  pT4 | 11  12  21  43 | 34  24  59  96 | 0.728 |
| Differentiation  Differentiated  Undifferentiated | 34  53 | 85  128 | 1.000 |
| Lymphatic involvement  Absent  Present | 10  77 | 31  182 | 0.128 |
| Vessel invasion  Absent  Present | 20  67 | 81  132 | 0.015 |
| Lymph node metastasis  Absent  Present | 18  69 | 74  139 | 0.012 |
| Peritoneal lavage cytology  Negative  Positive | 68  19 | 182  31 | 0.128 |
| UICC Stage  I  II  III  IV | 7  24  27  29 | 43  47  82  41 | 0.005 |

*ETNK2*, ethanolamine kinase 2; UICC, Union for International Cancer Control.

**Table S3.** Predictive factors associated with hepatic metastasis/recurrence for 300 patients with gastric cancer

| **Variables** | | **H/ H-rec (-)** | **H/ H-rec (+)** | **Univariate** | | | **Multivariate** | | |
| --- | --- | --- | --- | --- | --- | --- | --- | --- | --- |
|  |  |  |  | **OR** | ***P*** | **OR** | | **95%CI** | ***P*** |
| Age | < 65 year  ≥ 65 year | 118  155 | 7  20 | 2.18 | 0.088 |  | |  |  |
| Gender | Male  Female | 192  81 | 24  3 | 3.37 | 0.050 | 2.29 | | 0.62 - 8.32 | 0.208 |
| Carcinoembryonic antigen | ≤ 5 ng/ml  > 5 ng/ml | 224  47 | 15  12 | 3.81 | 0.001 | 2.39 | | 0.98 - 5.83 | 0.055 |
| Carbohydrate antigen 19-9 | ≤ 37 IU/ml  > 37 IU/ml | 213  55 | 19  8 | 1.63 | 0.275 |  | |  |  |
| Tumor location | Lower third  Others | 97  176 | 12  15 | 1.45 | 0.360 |  | |  |  |
| Tumor size | < 60 mm  ≥ 60 mm | 164  109 | 14  13 | 1.40 | 0.408 |  | |  |  |
| Macroscopic type | Borrmann type 4/5  Others | 40  233 | 1  26 | 0.22 | 0.148 |  | |  |  |
| Tumor depth | pT1-3  pT4 | 146  127 | 15  12 | 0.92 | 0.837 |  | |  |  |
| Tumor differentiation | Differentiated  Undifferentiated | 105  168 | 14  13 | 1.72 | 0.085 |  | |  |  |
| Lymphatic involvement | Absent  Present | 40  233 | 1  26 | 4.46 | 0.148 |  | |  |  |
| Vessel invasion | Absent  Present | 99  174 | 2  25 | 7.11 | 0.009 | 4.44 | | 0.95 - 20.7 | 0.058 |
| Infiltrative growth | Invasive  Expansive | 109  164 | 3  24 | 5.32 | 0.007 | 4.72 | | 1.33 - 16.7 | 0.016 |
| Lymph node metastasis | Absent  Present | 89  184 | 3  24 | 3.87 | 0.031 | 2.25 | | 0.59 - 8.51 | 0.232 |
| Peritoneal lavage cytology | Negative  Positive | 226  47 | 24  3 | 0.60 | 0.421 |  | |  |  |
| *ETNK2* expression | High  Low | 72  201 | 15  12 | 3.49 | 0.008 | 2.50 | | 1.05 - 5.95 | 0.038 |

*ETNK2*, ethanolamine kinase 2; H/ H-rec, hepatic metastasis/recurrence; OR, odds ratio; CI, confidence interval.

**Supplementary Figure Legends**

**Figure S1.** (a) Detection of an *ETNK2*-specific cleavage product in agarose gel electrophoresis for generation of the stable *ETNK2* KO cell lines. Indel, insertions and deletions. (b) Cell adhesion assays of untransfected and *ETNK2* KO MKN1 cells incubated with the indicated extracellular matrix proteins. BSA served as a negative control. (c) Cell cycle analysis of untransfected and *ETNK2* KO MKN1 cells. (d) Receiver operating characteristic curve analysis of the ability of *ETNK2* mRNA expression level to predict recurrence after curative gastrectomy for GC. The AUC (0.553) and cut-off value used for patient stratification (0.006) are shown. (e) Kaplan–Meier disease-free survival curves for patients with Stage II/III GC in the institutional and validation cohorts. (f) Representative images of IHC staining of GC tissues classified with negative, weak, or strong staining intensity for ETNK2 protein.


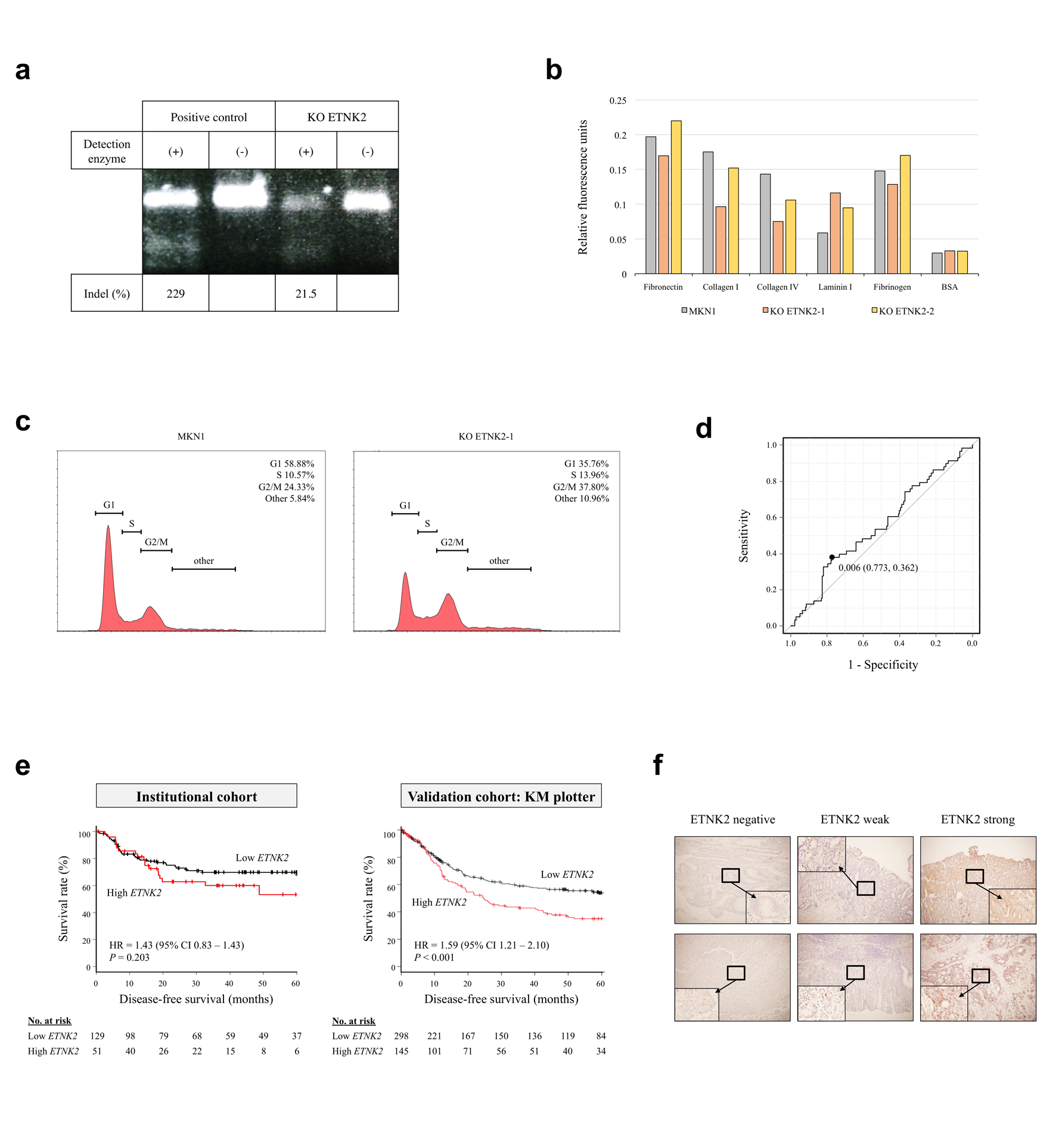

Supplement: Supplementary file 1 — Supplementary Materials [file 41416_2021_1271_MOESM1_ESM.doc]
